# Genetic mutations in Parkinson’s disease: screening of a selected population from North-Eastern Italy

**DOI:** 10.1007/s10072-024-07690-7

**Published:** 2024-07-22

**Authors:** Giulia Bonato, Angelo Antonini, Francesca Pistonesi, Marta Campagnolo, Andrea Guerra, Roberta Biundo, Manuela Pilleri, Cinzia Bertolin, Leonardo Salviati, Miryam Carecchio

**Affiliations:** 1 Parkinson and Movement Disorders Unit, Centre for Rare Neurological Diseases (ERN-RND), Department of Neuroscience, University of Padova, Via Giustiniani 2, 35128 Padua, Italy; 2 Center for Neurodegenerative Disease Research (CESNE), University of Padova, Padua, Italy; 3 Department of General Psychology, University of Padova, Padua, Italy; 4 Parkinson Institute, ASST G. Pini-CTO, Milan, Italy; 5 Department of Woman and Children’s Health, Genetic Unit, University of Padova, Padua, Italy

**Keywords:** Parkinson, Genetics, GBA, NGS

## Abstract

**Background:**

Parkinson’s disease (PD) is a progressive neurodegenerative disorder with a multifactorial pathogenesis. Several genetic variants increase the risk of PD and about 5–10% of cases are monogenic. This study aims to define the genetic bases and clinical features of PD in a cohort of patients from Northeastern Italy, a peculiar geographical area previously not included in genetic screenings.

**Methods:**

Using an NGS multigenic panel, 218 PD patients were tested based on age at onset, family history and development of atypical features.

**Results:**

A total of 133 genetic variants were found in 103 patients. Monogenic PD was diagnosed in 43 patients (20% of the cohort); 28 (12.8%) carried mutations in *GBA1*, 10 in *LRRK2* (4.6%) and 5 in *PRKN* (2.3%). In 17% of patients the genetic defect remained of uncertain interpretation. The selection criterion “age of onset < 55 years” was a significant predictor of a positive genetic test (OR 3.8, p 0.0037). *GBA1* patients showed more severe symptoms and a higher burden of motor and non-motor complications compared to negative patients (dyskinesias OR 3, sleep disturbances OR 2.8, cognitive deficits OR 3.6; p < 0.05), with greater autonomic dysfunction (COMPASS-31 score 34.1 vs 20.2, p 0.03).

**Conclusions:**

Applying simple clinical criteria for genetic testing allows to increase the probability to identify patients with monogenic PD and better allocate resources. This process is critical to widen the understanding of disease mechanisms and to increase the individuation of patients potentially benefitting from future disease-modifying therapies.

**Supplementary Information:**

The online version contains supplementary material available at 10.1007/s10072-024-07690-7.

## Introduction

Parkinson’s Disease (PD) is the second most common neurodegenerative disorder, with a wide spectrum of symptoms and variable response to dopaminergic therapy [1, 2]. Over the last 20 years, genetics has become increasingly relevant in understanding the biological mechanisms underlying PD etiology and progression. The use of Next Generation Sequencing (NGS) techniques and Genome Wide Association Studies (GWAS) has yielded more than 90 genomic loci influencing the risk of developing PD [3–5]. Monogenic PD forms represent about 5–10% of PD cases in previous unselected case series in Caucasians and up to 15% in more recent studies [6, 7]. The best characterized causative genes are *LRRK2*, the most common cause of autosomal dominant (AD) PD worldwide, *PRKN*, more common in younger patients with autosomal recessive (AR) inheritance, *PINK1*, *DJ1* and *SNCA* [8–10]. Mutations in the glucocerebrosidase (*GBA1*) gene, encoding a lysosomal enzyme involved in sphingolipid degradation, are now recognized as the main genetic risk factor for PD and as a negative clinical prognostic factor [11–13]. The prevalence of mutations in different PD-related genes varies considerably depending on patients’ ethnicity and age [8].

Northeastern Italy includes three different regions (Veneto, Trentino-Alto Adige and Friuli Venezia Giulia) with a population of approximately 11.5 million inhabitants and a vast mountain territory; historically and genetically, it holds some peculiarities, since it was the last region to join the Italian Reign in 1866 and it was politically linked to Central-Eastern Europe at the time of the Venetian Republic and later under the Austro-Hungarian Empire. This area of Italy has not been included in previous genetic studies on PD genetics, with the exception of South Tyrol [14], a small region bordering Austria with German-language predominance; thus, no genetic screenings of the Italian speaking population of this area have been performed so far.

Our study is aimed at: 1) screening a cohort of PD patients in a restricted geographical area of Italy on the basis of simple clinical criteria by an extensive NGS gene panel, to implement diagnostic yield; 2) estimating the prevalence of *GBA1* mutations and mendelian forms of PD in two movement disorders centers in North-Eastern Italy; 3) evaluating phenotypic features of patients with monogenic-PD.

## Materials and methods

Patients were recruited from two of the main tertiary referral centers specialized in movement disorders in North-Eastern Italy: the movement disorders unit of Padua University Hospital and Villa Margherita Clinic in Vicenza. Patients with a diagnosis of PD seen between 2017 and 2022 were included in the study. Genetic analysis was carried out in subjects fulfilling at least one of the following criteria: 1) onset of motor symptoms before 55 years of age; 2) a positive family history of movement disorders and/or cognitive decline (at least one relative of any degree); 3) development of cognitive and behavioral disturbances within the first 5 years after the diagnosis.

A total of 218 patients underwent genetic testing out of more than 1000 PD patients followed at both institutions; all the selected patients signed a standardized informed consent to undergo genetic analyses.

DNA was extracted from peripheral blood cells and analyzed at the Molecular Genetics Unit of Padua University by NGS sequencing using Illumina NextSeq550 or Illumina MiniSeq sequencers and an Agilent Sureselect custom kit. Reference genome was Genome Reference Consortium Human Build 37 (hg19).

Genetic analysis was carried out using a custom gene panel which targeted 155 genes associated with movement disorders (MD), including all genes currently recognized as PD-related and classified as PARK-n according to current nomenclature, as well as parkinsonism-related genes (that can sometimes manifest as PD-phenocopies at onset); research genes pending confirmation in PD were also analyzed. Diagnostic set for PD included *SNCA, LRRK2, GBA1, PRKN, PINK1, DJ1, VPS13C, SYNJ1, VPS35, ATP13A2, DNAJC6, FBXO7, PLA2G6, CHCHD2;* parkinsonism genes with a possible role in PD susceptibility or phenocopies included *GRN, MAPT, DCTN1, NPC1, NPC2, POLG, GCH1, ATP1A3;* research PD genes included *CSMD1, LRP10, TWNK, EIF4G1, DNACJ13, UCHL1*. The complete list of genes is available upon request.

The target region for sequencing comprised the entire coding region ± 10 nucleotides of each gene and was covered with a sequencing depth at least 20x for over 99% of the target. The coverage was satisfactory even for challenging genes like *GBA1*, in which the *GBA1LP* pseudogene interferes whit sequencing, and the number of reads uniquely mapping to *GBA1* reads was always above 20 for the entire target (Supplementary Figure S1). Bioinformatics CNV analysis [15] was performed in all cases, using the Agilent SureCall software with the “Default Haloplex Copy-Number Method” [16]. The *GBA1* gene was manually curated using IGV software [17]. To deeply assess the coverage quality of our customized gene panel, we compared whole exome and clinical exome analyses from a subset of patients included in this study using Illumina Exome panel (45 Mb) or the TruSight ONE expanded kits as previously described [16]. Interestingly, when we compared the performance of this custom panel with that of Illumina Trusight One Clinical exome kit we noted that the coverage was much lower in the latter and, most importantly, that the common p.Asp409Ser (N370S) variant was often called with suboptimal quality (and would have been missed applying strict filtering criteria) (Supplementary Figure S2).

Patients with onset before age 50, and those with single variants in recessive genes included in the kit were also studied by MLPA. MLPA analysis was performed using MRC Holland Probemix p051/p052 kit, which targets *SNCA, PRKN, UCHL1, PINK1, DJ1, ATP13A2, LRRK2* and *GCH1* genes, according to the manufacturer’s protocols.

Analysis was performed using the Variant Studio 3.0 software. We filtered out variants with read depth < 10x or alternate allele variant frequency < 20% and known artefacts from our internal database. We also excluded variants with MAF > 1% in GnomAD 2.1. Agilent SureCall software sets the maximum quality score at 255, but, given the small number of variants, we curated manually all relevant variants using IGV software independently of the quality score. Variants were then interpreted based on allele frequency (MAF in GnomAD 2.1), literature search, prediction tools (including Sift and Polyphen), and clinical features, and classified according to ACMG guidelines. For diagnostic purposes, in AD genes we considered and reported variants classified in ClinVar as Pathogenic or Likely pathogenic, or with MAF < 1:5000. For AR genes we considered biallelic variants for diagnosis.

Patients were assessed clinically: motor symptoms were scored by the Movement Disorder Society-Unified Parkinson's Disease Rating Scale III (MDS-UPDRS -III) scale and the Hoehn & Yahr (H&Y) stage [18], global cognitive functions with Mini-Mental State Examination (MMSE) and Montreal Cognitive Assessment (MoCA) scales [19]; medical and family history was collected. A subset of 92 patients (47 negative and 45 with genetic variants) underwent an extensive neuropsychological assessment including at least 2 tests for each of the 5 cognitive domains (executive, attention/working memory, visuospatial, memory and language abilities), a behavioral screening (assessing depression, anxiety, apathy, impulse control disorders -ICD- and impulsivity), functional autonomy and quality of life (for tests details see Supplementary Table I). Patients were evaluated in ‘on’ medication state. MMSE and MoCA total scores were adjusted for age and education, and z scores were calculated for all the cognitive tests according to Italian normative data. The neuropsychological evaluation was administered by trained neuropsychologists, blinded to genetic profile. Dementia and mild cognitive impairment (MCI) were diagnosed according to the published MDS Level II PD criteria [19].

The scale Composite Autonomic Symptom Score-31 (COMPASS-31) [20] was administered to patients with *GBA1* mutations and to a negative control group of PD patients with similar demographic features, to investigate autonomic disturbances.

Statistical analysis was conducted with Chi-squared or Fisher test for qualitative data, Mann–Whitney or Kruskall-Wallis test for quantitative data, as appropriately needed (GraphPad Prism Software Version 9.4.1).

## Results

218 patients, 132 males (60.5%) and 86 females (39.4%) were included. A detailed cohort description is shown in Table 1. Mean age at motor onset was 50.8 years, with 37% of patients presenting in the age range 41–50 years, and 15% with onset under 40 years of age. The most frequent symptoms at onset were bradykinesia and/or rigidity (51%) whereas cognitive or psychiatric symptoms at onset were present in 7% of the cohort. Family history was positive in 33% of the subjects for movement disorders and in 4% for cognitive decline of various degrees of severity. Mean disease duration was 8.8 years with a wide range (1–45 years); the majority of patients had moderate symptoms, with more than half of the subjects showing motor complications (mean L-Dopa Daily Dose -LEDD- 717 mg/die, UPDRS-III 22.5, H&Y 2.2) and complaining of neuropsychiatric disturbances (such as anxiety, depression, impulse control disorder) and/or cognitive deficits; 37% of them were treated with advanced therapies (Deep Brain Stimulation -DBS- in 23% of cases and infusion therapies -LCIG- in 15%).

**Table I:** Clinical features of the cohort and main genetic subgroups; data were used for genotype-phenotype correlation study and comparison between genetic patients and controls

	Cohort (218 Pt)	Negative group (115 Pt)	GBA (28 Pt)	PARK2 (5 Pt)	LRRK2 (10 Pt)	CSMD1 (13 Pt)
General demographics						
M:F	1,5	1,3	1,5	4	9	1,2
Mean age at onset (y-sd)	50,8 (± 10,7)	52 (± 9,8)	47,8 (± 8,5)	31,2 (± 19,7)	51,0 (± 6,1)	60,0 (± 10,7)
Mean age last evaluation (y)	59,7 (± 9,7)	60,7 (± 9,9)	57,4 (± 6,0)	49,8 (± 7,9)	59,8 (± 6,3)	66,4 (± 10,7)
Mean disease duration (y)	8,8 (± 6,6)	8,6 (± 6,1)	9,6 (± 5,5)	18,8 (± 17,1)	8,8 (± 4,5)	6,4 (± 4,4)
Positive family history (% Pt)	37%	35%	25%	40%	50%	62%
Onset: % Pt (*n*)						
Rigidity	9% (20)	11% (13)	7% (2)	0% (0)	10% (1)	0% (0)
Bradykinesia	31% (70)	28% (33)	32% (9)	20% (1)	50% (5)	31% (4)
B/R	11% (24)	9% (11)	21% (6)	20% (1)	10% (1)	0% (0)
Isolated tremor	30% (67)	36% (43)	14% (4)	20% (1)	20% (2)	38% (5)
Mixed TBR	14% (31)	11% (13)	18% (5)	40% (2)	0% (0)	15% (2)
Cognitive slowing	2% (4)	1% (1)	4% (1)	0% (0)	0% (0)	8% (1)
Major psychiatric disturbance	5% (11)	3% (4)	4% (1)	0% (0)	10% (1)	8% (1)
Symptoms and complications at last evaluation: % Pt (*n*)						
Falls	27% (59)	26% (30)	43% (12)	20% (1)	50% (5)	23% (3)
Aids	18% (39)	18% (21)	21% (6)	20% (1)	10% (1)	23% (3)
Incontinence	35% (76)	40% (46)	39% (11)	20% (1)	10% (1)	31% (4)
Dyskinesia	45% (98)	42% (48)	68% (19)	80% (4)	80% (8)	31% (4)
Dystonia	35% (76)	34% (39)	43% (12)	40% (2)	50% (5)	8% (1)
Fluctuations	56% (121)	56% (64)	75% (21)	60% (3)	70% (7)	38% (5)
ICD	24% (53)	20% (23)	36% (10)	60% (3)	10% (1)	31% (4)
Hallucinations	17% (36)	14% (16)	29% (8)	20% (1)	0% (0)	23% (3)
Sleep disturbances	55% (120)	51% (59)	75% (21)	40% (2)	20% (2)	62% (8)
RBD	18% (39)	17% (19)	25% (7)	20% (1)	20% (2)	8% (1)
Cognitive deficits	39% (84)	33% (38)	64% (18)	80% (4)	20% (2)	46% (6)
MCI	27% (58)	24% (28)	39% (11)	80% (4)	20% (2)	31% (4)
Dementia	7% (15)	5% (6)	18% (5)	0% (0)	0% (0)	15% (2)
Psychiatric features	60% (131)	57% (66)	68% (19)	60% (3)	40% (4)	62% (8)
Evaluations scales						
MMSE (mean)	25,1 (± 4,0)	25,6 (± 3,5)	24,0 (± 4,4)	26,4 (± 2,1)	27,5 (± 2,1)	25,1 (± 5,6)
MoCa (mean)	21,7 (± 5,0)	22,7 (± 4,5)	19,9 (± 5,2)	19,9 (± 3,9)	22,5 (± 2,5)	21,2 (± 6,2)
UPDRS (mean)	22,5 (± 14,6)	21,4 (± 13,5)	24,2 (± 12,5)	19,6 (± 3,5)	18,4 (± 8,7)	29,0 (± 18,4)
H&Y (mean)	2,2 (± 0,9)	2,1 (± 0,9)	2,4 (± 0.8)	2,6 (± 0,2)	2,0 (± 0,2)	2,1 (± 0,9)
Therapy						
DBS - % Pt (*n*)	21% (45)	16% (18)	39% (11)	60% (3)	40% (4)	0% (0)
PEG-J - % Pt (*n*)	12% (27)	17% (19)	0% (0)	0% (0)	10% (1)	23% (3)
DBS+PEG-J - % Pt (*n*)	2% (4)	1% (1)	11% (3)	0% (0)	0% (0)	0% (0)
Only oral therapy - % Pt (*n*)	64% (139)	66% (76)	46% (13)	40% (2)	50% (5)	77% (10)
Apomorphine+/-DBS	2% (3)	1% (1)	4% (1)	0% (0)	0% (0)	0% (0)
LEDD mean (mg/24h)	717,7 (± 472,5)	778,0 (± 554,7)	674,9 (± 312,9)	731,0 (± 419,0)	552,7 (± 301,9)	566,5 (± 412,9)

*Pt*, patients; *n*, number of patients; *%Pt*, percentage of patients; *M*, male; *F*, female; *y*, years; B, bradykinesia; *R*, rigidity; *T*, tremor; *ICD*, impulse control disorder; *RBD*, Rem-sleep behaviour disorder; *MCI*, mild cognitive impairement; *MMSE*, MiniMental State Examination; *MoCA*, Montreal Cognitive Assessment; *UPDRS*, Unified Parkinson’s Disease Rating Scale; *H & Y*, Hoehn & Yahr scale; *DBS*, deep brain stimulation; *PEG-J*, Duodopa gel jejunal infusion therapy; *LEDD*, Levodopa equivalent daily dose

We found 103/218 subjects (47%) carrying at least one variant in genes related to movement disorders; variants in PD/parkinsonism genes were found in 79 patients, in PD research genes were found in 20 (4 of which in combination with PD genes) and 20 patients had more than one variant in multiple genes.

**Fig. 1:**
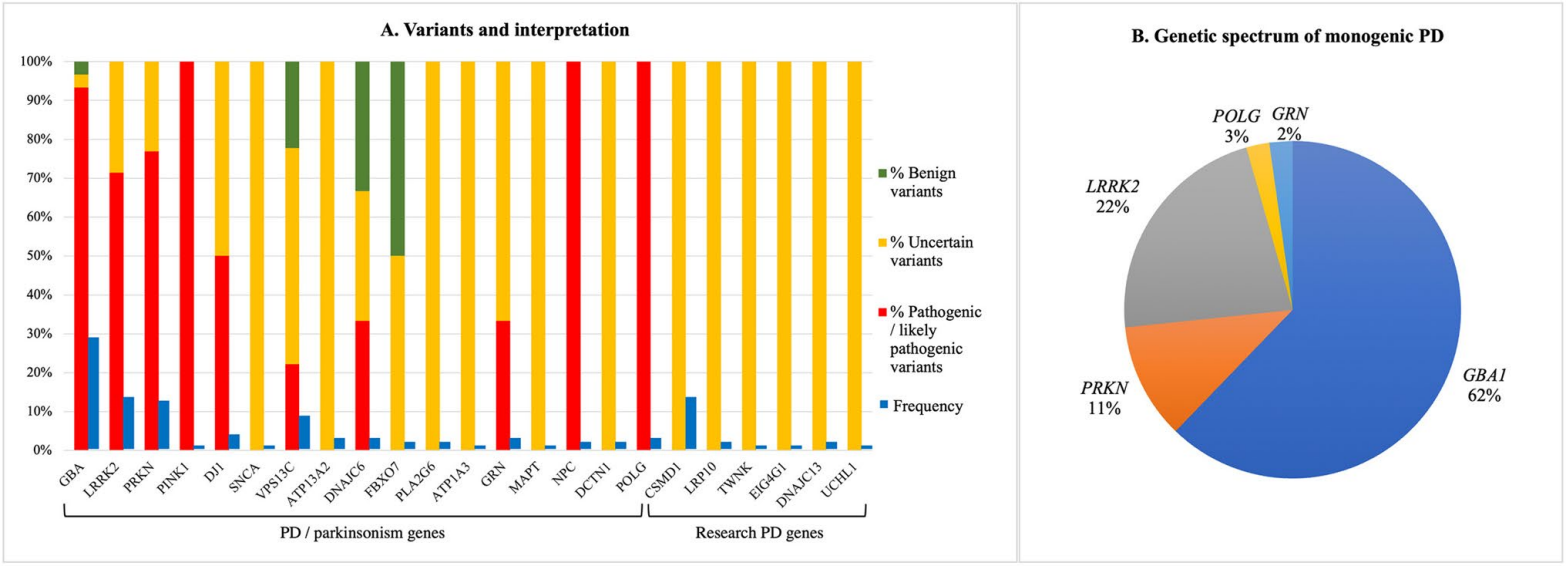
**A** PD, parkinsonism and research genes found in the study: frequency and classification of the variants in each gene as pathogenic, VUS or benign according to ACMG classification; **B** Genetic spectrum of patients with a definite diagnosis of monogenic PD

A total of 133 variants were found (complete list in Supplementary Table II); 94 were in PD/parkinsonism genes (72 were in “classic” PD genes *GBA1, PRKN, PINK1, DJ1, SNCA, LRRK2, VPS13C*), 21 in “research PD genes”, and 18 in genes related to other movement disorders. According to ACMG classification, 47% were classified as pathogenic or likely pathogenic mutations (ACMG class 4–5), 48% were variants of uncertain significance (VUS, ACMG class 3) and 5% were considered likely benign variants and were not considered for diagnosis (Fig. 1A). Variants were mainly of a missense type (80%), whereas 12% were frameshift or truncating variants, 5% affected splicing and only 2% were small in frame or exons deletions.

*GBA1* had the highest mutational frequency (30/133 variants, found in 27% of the 103 patients carrying genetic variants), followed by *PRKN* (13/133, in 10.6% of patients with variants), *LRRK2* (14/133, in 13.5% of patients with variants) and *CSMD1* (14/133), whereas other recessive genes such as *DJ1* and *PINK1* were rarely found (4/133 and 1/133) (Fig. 1A). One patient carried a *PRKN* deletion identified by CNV analysis and then confirmed by MLPA.

Based on the interpretation of genetic variants and on patients’ clinical features, a final diagnosis of monogenic PD was formulated in 43 patients (41.7% of patients carrying any genetic variant and 20% of the whole cohort, Table 2) with a significant prevalence of mutations in *GBA1* (28/103 subjects, 12.8% of the whole cohort), followed by *LRRK2* (10/103 patients, 9.7%), *PRKN* (5/103 patients, 4.8%). Additionally, one patient had biallelic *POLG* mutations leading to tremor-dominant parkinsonism in her fifth decade with altered DAT-Scan, associated with palpebral ptosis and later in life with myopathy and peripheral neuropathy; one patient carried a pathogenic *GRN* mutation and received a diagnosis of FTDP (frontotemporal dementia-parkinsonism) with a clinical presentation consistent with a PD phenocopy (Fig. 1B, Supplementary Table III). In 23 patients (10.5% of the cohort, 22.3% of patients with variants) it was not possible to determine a pathogenic role of genetic variants found in PD genes; these cases included single previously unreported VUS in dominant genes (4 in *LRRK2*) and monoallelic variants in recessive genes (6 *PRKN,* 1 *PINK1,* 4 *DJ1, 6 VPS13C, 3 ATP13A2,* 1 *DNAJC6,* 2 *PLA2G6,* 50% likely pathogenic); 7 patients had multiple variants in more than one PD gene. Additionally, 8 subjects carried single variants in parkinsonism-related genes with a possible role in PD risk and predisposition. The presence of VUS in research genes was detected in 17 subjects.

**Table 2:** Genetic diagnosis following genetic test

Diagnosis	*n*. patients	% var+	% cohort
Monogenic PD	45	44%	21%
Uncertain diagnosis (variants in PD genes)	23	22%	10.5%
Negative - Uncertain role (parkinsonism-related genes)	8	7.7%	3.7%
Negative - Uncertain role (research genes)	17	16.5%	7.8%
Negative - Non-PD-related or benign variant	10	9.7%	4.6%
Total subjects with any variant (var+)	103		47%
Negative test, no variants found	115		53%
Total cohort	218		100%

*n* number; % var+: percentage over subjects with any genetic variant (103); % cohort: percentage over whole cohort (218 subjects)

Among selection criteria used, age at onset under 55 years proved to perform better in predicting patients’ genetic status in monogenic PD: 86% of patients with a positive genetic test, in fact, had disease onset under 55 years vs 62% of negative subjects (OR 3.8, 95% IC 1.5–9.3; p 0.0037); 25.8% of subjects with symptom onset ≤ 55 years received a genetic diagnosis vs 8.5% of those with later onset, who only had mutations in *GBA1* (4 subjects) and *LRRK2* (2 patients); 1 subject with pathogenic biallelic *PRKN* mutations had symptoms onset at 55 years. Conversely, a positive family history per se was not a good predictor of a positive genetic test (33.3% in monogenic PD patients vs 34.8% in negative subjects, p 0.9).

A genotype–phenotype correlation study was conducted comparing different genetic groups and 115 patients without genetic variants.

**Fig. 2:**
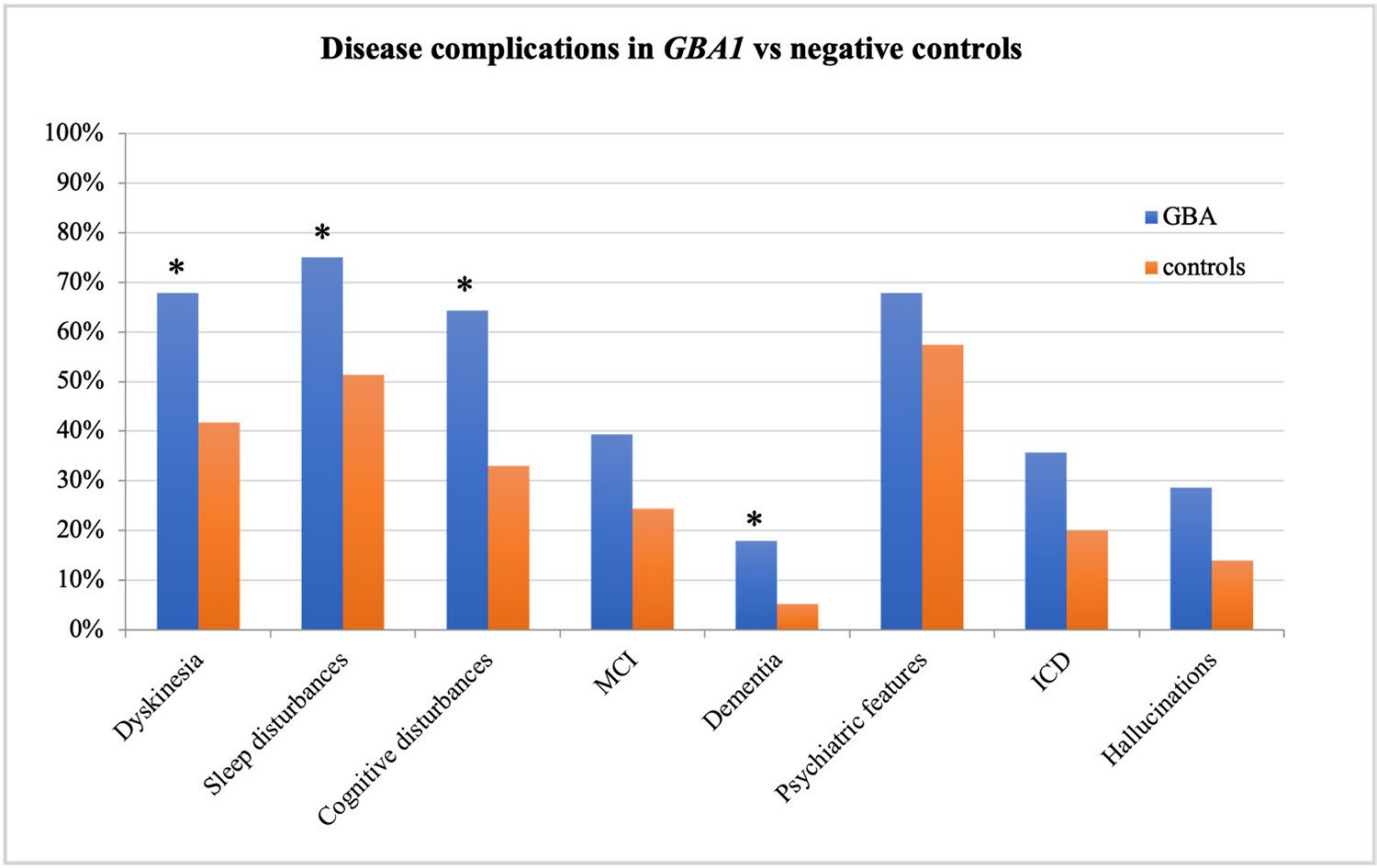
Main clinical features of *GBA1*-PD patients compared to negative controls; * indicates statistically significant differences between the two groups

Mutations in *GBA1* were the most common finding: 29 pathogenic variants were found in 28/218 patients (12.8% of the cohort, 62% of positive diagnosis), with biallelic mutations in one patient consistent with a diagnosis of Gaucher disease associated with PD. The most common *GBA1* variants in our cohort were the p.Asn409Ser (N370S) and the p.Asp448His (D409H) in 11 and 5 subjects, respectively, whereas the p.Leu483Pro (L444P) and the p.Glu365Lys (E326K) were isolated findings as well as other less frequent variants (Supplementary Table IV). 70% of *GBA1* patients developed symptoms before age 50, with mean age at onset of 47.8 years; a positive family history was reported in 25% of these subjects; onset with isolated rest tremor was infrequent (14% vs 36% in negative controls, p 0.02). *GBA1*-PD patients had a higher burden of motor complications than negative PD patients with comparable disease duration; these included dyskinesia (68% vs 42%, p 0.01, OR 3,95%IC 1.3–8.8), sleep problems including Rem Behavior Disorder RBD (75% vs 51%, OR 2.8, 95%IC 1.1–7.1, p 0.03) and any cognitive disturbances (64% vs 33%, OR 3.6, 95%IC 1.5–8.7 p 0.004). *GBA1*-PD showed a significant lower mean MoCA score, and higher percentage of dementia compared to negative controls (p 0.04) (Fig. 2, Table 1). There was no significant difference in the prevalence of hallucinations, ICD and psychiatric disturbances (p 0.07) although these were a frequent trend in *GBA1*-PD compared to the negative control group and idiopathic late onset PD; accordingly, *GBA1*-PD patients necessitated more of advanced therapy approaches (14/28 *GBA1*-PD patients underwent DBS and 4 were treated with LCIG).

Dysautonomia was tested using the COMPASS-31 scale in 16 *GBA1*-PD subjects and 18 randomized PD patients with negative genetic test, with comparable demographic data, disease onset, duration and LEDD; mean total score was significantly higher in *GBA1* patients (34.1 vs 20.2, p 0.03), indicating a higher burden of dysautonomia.

We could not detect clear gender differences in GBA subgroup due to the sample size, but females showed a higher tendency to present with tremor dominant PD, with depression as the main neuropsychiatric feature (64% vs 30%, p 0.1), a higher rate of falls (64% vs 29%, p0.1) and motor complications, especially dyskinesias (91% vs 53%, p 0.049); no relevant differences could be detected in rates of cognitive disturbances and dysautonomia; men had higher rates of sleep disturbances (82% vs 64%, p 0.3). Similarly, no differences could be detected in severity of mutations.

14 patients had variants in the *LRRK2* gene, of which 10 pathogenic (4.5% of the cohort, 22% of positive diagnoses) and 4 classified as VUS; the p.Gly2019Ser (G2019S) was found only in 4 subjects; clinically, patients had onset over 50 years of age, with less frequent cognitive manifestations (including ICD and hallucinations) and sleep disturbances, and higher functional autonomy with less aids even with lower L-Dopa therapy doses (see Table 1 for clinical details).

5 patients carried *PRKN* biallelic mutations (11% of monogenic PD, 2.3% of the cohort) and 6 subjects carried single pathogenic variants (in 2 subjects in association with VUS in other PD related genes); clinically, these patients had younger disease onset (mean 31.2 years, p < 0.05, with youngest onset at age 4 with clumsiness of gait and dystonia of the lower limbs due to *PRKN* homozygous mutation c.823C > T p.Arg275Trp [21]). Moreover, the percentage of motor fluctuations and cognitive-neuropsychiatric disturbances (especially impulsivity) were higher compared to PD negative patients and *GBA1*- and *LRKK2*-related PD, although the differences were not significant due to the small sample size (see Table 1). *PRKN* mutations were the most frequent cause of juvenile PD in our cohort (2/2 patients with disease onset < 20 years) and a significant cause of early onset disease (3/32 patients with onset < 40 years).

As for research genes, the most frequent findings were VUS in *CSMD1,* detected in13 patients (6%), but their clinical features did not differ significantly from negative controls (Table 1) and resembled idiopathic PD, with onset around 60 years of age and rare occurrence of dystonia and RBD (8%); there was a positive family history in 62% but segregation study in one family did not support a pathogenic role of the *CSMD1* variant found.

**Fig. 3:**
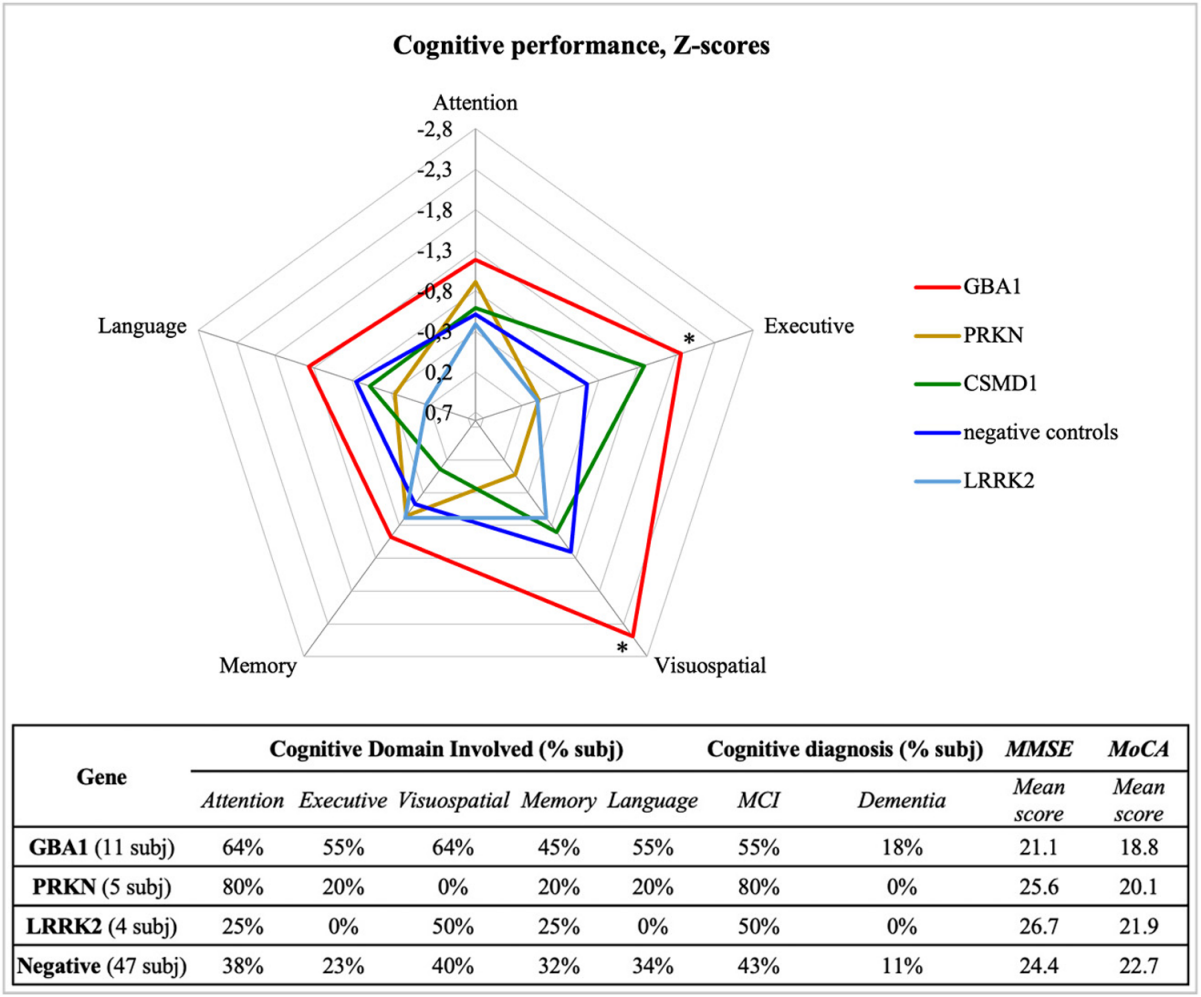
Results of cognitive assessment showing mean Z scores in the five cognitive domains in different genetic subgroups of patients (*indicates pathologic mean scores). The lower part of the image reports the percentage of patients with difficulties in the same cognitive domains

The complete cognitive evaluation, assessed in a subset of 92 patients (11 *GBA1*, 5 *PRKN*, 4 *LRRK2*, 47 negative controls, 25 VUS) showed pathological performance (at least -1.5 z score below appropriate norms) in *GBA1* patients in visuospatial and executive domains (in 64% and 55% respectively), and an overall tendency to lower mean z scores in each cognitive domain compared to negative controls and other monogenic PD forms. 80% of *PRKN* patients showed attention difficulties, whilst *LRRK2* patients had a lower cognitive burden and none were demented (Fig. 3).

## Discussion

In this study, we performed a genetic screening of a cohort of PD patients from Northeastern Italy, an area with some geographical and historical peculiarities that was not previously involved in genetic screening programs. We chose to employ a custom panel because of the significantly lower costs, the higher number of patients that can be studied simultaneously and the better coverage of critical genes compared to whole exome or clinical exome kits.

In our cohort, we found a higher prevalence of *GBA1* mutations compared with other frequent causes of monogenic PD such as *LRRK2* and *PRKN* [8, 9, 22].

In line with recent studies conducted on the Italian PD general population [13, 23], our data confirm a high mutational frequency of *GBA1* (12.8%) in Italian PD patients compared to older studies. *GBA1* mutations were also frequent in under-40 patients (13%), but with a lower percentage than previously shown by Petrucci et al. (20.4% in early onset PD, 14.3% in overall PD population) [13]; this discrepancy possibly being due to differences in geographical recruitment areas and selection criteria for genetic testing.

Our study confirms that genetic bases influence disease course; this is especially evident for patients with *GBA1*-PD [12, 13, 24], that in our cohort show a higher proportion of cognitive and psychiatric features, sleep and autonomic dysfunction as well as motor complications compared to other PD patients with or without identifiable mutations. This observation has important consequences on patients’ quality of life and possible future implications for therapy [25, 26], and it is especially relevant for autonomic dysfunction, which represents both a diagnostic red flag for *GBA1* and a challenge for disease management. The COMPASS-31 scale in our experience proved to be a quick and handy tool to screen PD patients for dysautonomic symptoms in a clinical setting.

In line with previous literature [11], the p.Asn409Ser (N370S) was the most frequent *GBA1* variant in our cohort, whereas the p.Leu483Pro (L444P), the second most common variant worldwide, and the p.Glu365Lys (E326K) were found in only one patient each, probably reflecting differences in the geographical background of single variants; moreover, we found higher frequencies of less common and non-canonical *GBA1* variants, thus confirming the need for a complete screening of *GBA1* gene with modern techniques, in order to obtain a correct diagnosis and prevalence estimation, as recently highlighted [13, 23].

*LRRK2* was the second most frequent finding in our cohort of monogenic PD; the frequency of the p.Gly2019Ser (G2019S) variant was 1.8% in our cohort, consistently with previous Italian data [27], but lower than other Mediterranean countries [22, 28] and more similar to continental Europe. A low genetic exchange with other Mediterranean areas and the previous belonging to the Austro-Hungarian empire could explain this difference as well as the selection of younger patients; in fact, the penetrance of *LRRK2* mutations is age-dependent and characterized by clinical manifestations similar to classic PD, with low cognitive burden and complications [28], as confirmed in our cohort.

The frequency of *PRKN* mutations was the main cause of juvenile onset PD and 2.3% of the entire cohort, with a peculiar case of childhood onset [21] and 2 cases of atypical onset over 50 years of age. Clinically, these *PRKN* cases confirm the presence of early neuropsychiatric features and younger onset in this genetic PD subtype [29, 30].

We could not detect biallelic mutations in other recessive genes, such as *PINK1* and *DJ1*, thus confirming their relative rarity.

Our data highlight that young age at onset, but not presence of family history for movement disorders, is associated with greater likelihood to find PD patients with positive genetic testing. We believe this finding enforces the need to offer genetic testing also to patients without a clear positive family history. The individuation of simple selection criteria to be used in clinical practice for genetic testing allows to better allocate resources and increase the diagnostic yield, detecting a higher proportion of subjects carrying pathogenic genetic variants who could benefit from future disease modifying treatments. This could also favor a correct timing for family counseling, allowing prompt information of young patients about the risk of disease transmission to offspring. However, the proportion of mutated subjects in older patients is non-negligible (8.5%), especially for dominant genes such as *GBA1* and *LRRK2*, having implications for family counseling and disease management, so the ideal goal would be to offer genetic testing to all PD patients at the time of diagnosis.

One of the challenges deriving from the use of NGS panels is the detection of an increasing number of VUS in PD/parkinsonism related genes and research genes whose pathogenetic role is debated. This includes numerous variants in *CSMD1* [31] which we found in 13 patients of our cohort. In one family we performed a segregation analysis that did not support the pathogenic role of the variant found. Likewise, mutations in *LRP10*, a gene rarely documented in PDD, PD and DLB [32], were seldom found in our cohort, with only 2 VUS found and no association with cognitive deficits. Genetic counselling can be quite problematic in those cases and a good collaboration between geneticists and clinicians with an expertise in PD genetics is warranted. Other challenges in data interpretation are due to the presence of monoallelic variants in PD recessive genes (representing the majority of uncertain cases in our cohort), whose role as PD risk factor is still uncertain [14, 33–36]. The presence of a second, unidentified intronic variant in these patients may explain their phenotype.

The role of VUS in genes usually related to other movement disorders or atypical parkinsonism is presently unknown. In our cohort, 9.2% of subjects had variants in multiple MD-related genes that could possibly modulate pathogenetic mechanisms and give rise to motor manifestations consistent with PD diagnostic criteria. In this regard, one patient carrying a pathogenic *GRN* mutation presented early onset of asymmetric bradykinesia and rigidity without cognitive or psychiatric manifestations after 3-year of follow-up. In clinical practice the role of multiple variants in single genes probably determines a higher cumulative disease risk to develop the disease outside the classical mendelian inheritance rules [4, 5, 37]. In all these cases, it is not possible to estimate the risk of transmission of the disease to offspring and genetic counselling cannot provide a reliable risk assessment. While acknowledging the challenges and constraints in interpreting VUS in PD, the application of NGS offers a valuable opportunity to expand the scope of etiological diagnoses, with the overarching goal of identifying prognostic factors and exploring targeted therapeutic interventions [38, 39].

In summary, there is a growing interest in leveraging biological markers, particularly genetic markers, for the comprehensive characterization of PD and the precise delineation of its progression and trajectories [2, 40]. Our findings suggest that distinct genotypes may correlate with diverse patterns of progression, clinical manifestations, and therapeutic requirements. Given the widespread availability of genetic screening at many institutions, we advocate for its early implementation, ideally at the time of diagnosis, for all PD patients. This not only will enhance diagnostic accuracy but will also contribute to a more refined prognosis and understanding of pathological processes.

## Supplementary Information

Below is the link to the electronic supplementary material.

Supplementary file1 (DOCX 720 KB)

## Data Availability

Additional data are available from the corresponding author on reasonable request.
