# Supplementary material for: Genetic mutations in Parkinson’s disease: screening of a selected population from North-Eastern Italy
Source: Neurol Sci. 2024 Jul 22;46(1):165–74. doi: 10.1007/s10072-024-07690-7 (PMC11698772; doi:10.1007/s10072-024-07690-7)
Supplement: Supplementary file 1 — Supplementary file1 (DOCX 720 KB) [file 10072_2024_7690_MOESM1_ESM.docx]

**Genetic mutations in Parkinson’s disease: screening of a selected population from North-Eastern Italy**

**Neurological Sciences**

Giulia Bonato^1,2^, Angelo Antonini^1,2^, Francesca Pistonesi^1,2,3^, Marta Campagnolo^1,2^, Andrea Guerra^1,2^, Roberta Biundo^1,2,3^, Manuela Pilleri^4^, Cinzia Bertolin^5^, Leonardo Salviati^5^, Miryam Carecchio^1,2^*

*1. Parkinson and Movement Disorders Unit, Centre for Rare Neurological Diseases (ERN-RND), Department of Neuroscience, University of Padova, Padova, Italy*

*2. Center for Neurodegenerative Disease Research (CESNE), University of Padova, Padova, Italy*

*3. Department of General Psychology, University of Padova, Padova, Italy*

*4. Parkinson Institute, ASST G. Pini-CTO, Milan, Milan, Italy*

*5. Department of Woman and Children’s health, Genetic Unit, University of Padova, Italy*

***Corresponding author:**

Miryam Carecchio, MD, PhD

Email: miryam.carecchio@unipd.it

**Supplementary Material**

**
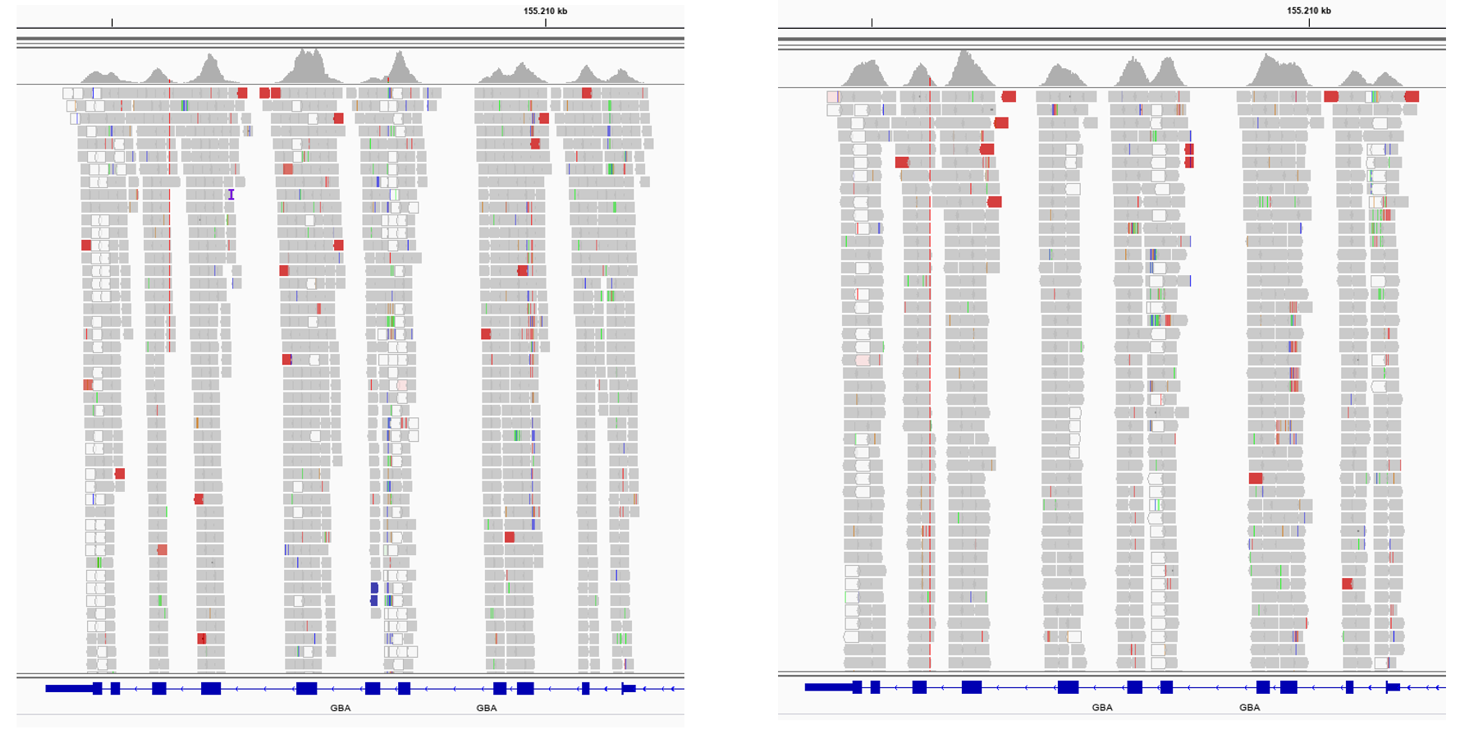
**

**Fig. S1:** The GBA gene analyzed with a WES panel (Left) or by the custom panel (Right). Average coverage on the target region was 89x for the WES and 284x for the panel.


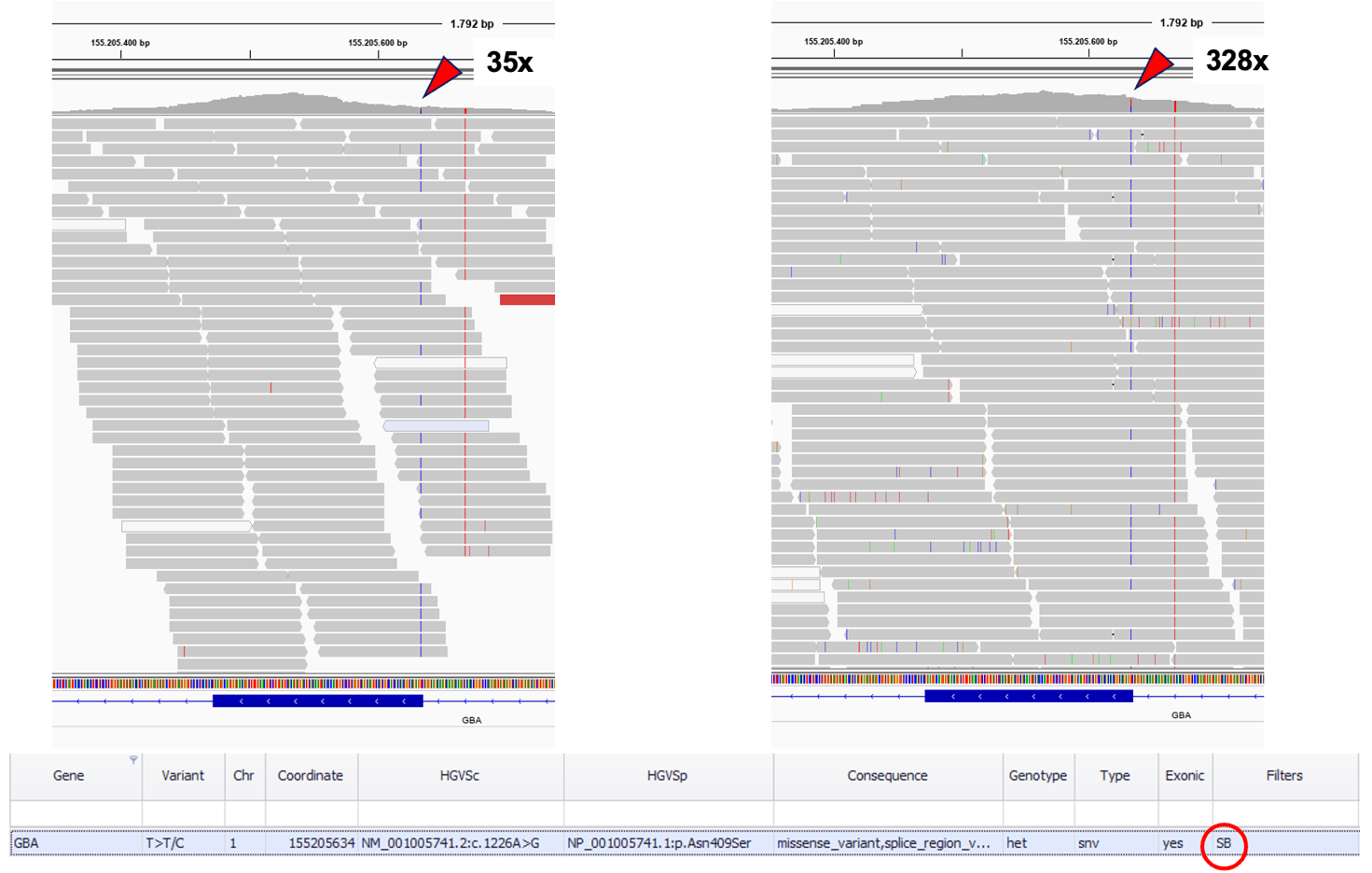


**Fig. S2:** (Left) suboptimal coverage of nucleotide position 1226 of the *GBA* gene using a Clinical exome panel causing «strand bias» compared to the same region analyzed by the custom panel (Right). The p.Asn409Ser was called but with strand bias (circle) as the majority of reads were mapping on the minus strand.

**Supplementary Table I:** List of neuropsychological tests administered to patients and corresponding references

.

| *Function/feature analyzed* | *Test* | *References* |
| --- | --- | --- |
| Global cognitive tests | Montreal Cognitive Assessment (MoCA) | Nasreddine 2005, Santangelo 2013 |
|  | Mini-Mental State Examination (MMSE) | Folstein 1975 |
| Executive domain | Stroop Color/Word test, error and time (STROOP) | Caffarra 2002 |
|  | Clock Drawing Test (CDT) | Caffarra 2002 |
|  | Phonemic fluency | Novelli 1986 a |
| Attention and working memory | Digit Span Sequencing (DSS) | Wechsler 2008 |
|  | Trail-Making-Test A, B and B-A (TMT-A, -B, B-A) | Giovagnoli 1996 |
|  | Symbol Digit Modalities Test (SDMT) | Nocentini 2006 |
| Visuospatial ability | Rey–Osterrieth complex figure (ROCF) | Caffarra 2002 |
|  | Benton’s Judgment of Line Orientation test | Gullett 2013 |
|  | Visual Object and Space Per- ception (VOSP) | Warrington and James 1991 |
| Memory | Wechsler Paired-Associates Test (WPAT) | Novelli 1986 b |
|  | ROCF delayed | Caffarra 2002 |
|  | Babcock story recall | Spinnler and Tognoni 1987 |
| Language ability | Semantic fluency | Novelli 1986 a |
|  | Boston naming test | Williams 1989 |
| Depression | Beck Depression Scale (BDI-II) | Beck 1996, Ghisi 2006 |
| Apathy | Starkstein’s Apathy Scale | Starkstein, 1992 |
| Anxiety | State–Trait Anxiety Inventory (STAI Y-1, Y-2) | Spielberger 1964, Pedrabissi 1989 |
| Impulsivity | Barratt Impulsiveness Scale (BIS-11) | Fossati 2011 |
| ICD | Questionnaire for Impulsive-Compulsive Disorders in Parkinson's Disease–Rating Scale (QUIP-RS) | Weintraub 2012 |
| Cognitive autonomy | Parkinson’s Disease Cognitive Functional Rating Scale (PD- CFRS) | Kulisevsky, 2013 |
| Quality of life | 8-item version of Parkinson’s disease quality of life (PDQ-8) | Jenkinson 1997 |
| Functional autonomy | Activities of daily living (ADL) | Katz, 1970 |
|  | Instrumental ADL (IADL) | Lawton, 1969 |

**Supplementary Table II:** Full list of variants found in the study, with absolute frequency, MAF and ACMG. Variants that were used for definite diagnosis are in bold letters.

| ***Gene*** | ***Variant*** | ***N*** | ***MAF*** | ***ACMG*** | ***ACMG Criteria*** |
| --- | --- | --- | --- | --- | --- |
| *APP* | NM_000484.3:c.1995G>C p.(Glu665Asp) | 1 | 1.19e-5 | 3 | PM2 |
| *ATP13A2* | NM_022089.2:c.2797G>A p.(Val933Ile) | 1 | 3.98e-5 | 3 | PM2 |
| *ATP13A2* | NM_022089.2:c.2978C>T p.(Pro993Leu) | 1 | 6.44e-5 | 3 | PM2 |
| *ATP13A2* | NM_022089.2:c.3409G>A p.(Val1137Met) | 1 | 2.29e-5 | 3 | PM2 |
| *ATP1A3* | NM_001256214.1:c.877C>T p.(His293Tyr) | 1 | 0 | 4 | PM1, PP2, PM2, PP3 |
| *ATP7B* | NM_000053.4:c.3955C>T p.(Arg1319*) | 1 | 7.12e-5 | 5 | PM3, PVS1, PM2, PP5 |
| *CSMD1* | NM_033225.5:c.10514C>G p.(Ala3505Gly) | 1 | 0 | 3 | PM2, BP4 |
| *CSMD1* | NM_033225.5:c.10201+4G>C | 1 | 1.22e-5 | 3 | PM2, BP4 |
| *CSMD1* | NM_033225.5:c.7391G>A p.(Arg2464Gln) | 1 | 1.60e-5 | 3 | PM2 |
| *CSMD1* | NM_033225.5:c.7460C>T p.(Thr2487Met) | 1 | 2.50e-5 | 3 | PM2, BP4 |
| *CSMD1* | NM_033225.5:c.5201C>G p.(Pro1734Arg) | 1 | 1.48e-5 | 3 | PM2 |
| *CSMD1* | NM_033225.5:c.2198C>A p.(Ser733Tyr) | 1 | 8.03e-6 | 3 | PM2 |
| *CSMD1* | NM_033225.5:c.8246C>T p.(Thr2749Ile) | 1 | 0 | 3 | PM2 |
| *CSMD1* | NM_033225.5:c.10202-4A>T | 1 | 0 | 3 | PM2 |
| *CSMD1* | NM_033225.5:c.9190A>C p.(Asn3064His) | 1 | 0 | 3 | PM2 |
| *CSMD1* | NM_033225.5:c.8498T>C p.(Leu2833Ser) | 2 | 0 | 3 | PM2, PP3 |
| *CSMD1* | NM_004082.4:c.1400C>T p.(Thr467Met) | 1 | 1.22e-5 | 3 | PM2 |
| *CSMD1* | NM_033225.5:c.7699C>T p.(His2567Tyr) | 1 | 8.51e-6 | 3 | PM2 |
| *CSMD1* | NM_033225.5:c.2153C>T p.(Ser718Leu) | 1 | 2.82e-5 | 3 | PM2 |
| *DCTN1* | NM_004082.4:c.2746C>T p.(Arg916Trp) | 1 | 1.19e-5 | 3 | PM2 |
| *DCTN1* | NM_004082.4:c.2606T>A p.(Leu869Gln) | 1 | 0 | 3 | PM2, PP3 |
| *DHDDS* | NM_205861.3:c.188G>A p.(Arg63Gln) | 1 | 7.97e-6 | 3 | PM2 |
| *DHX30* | NM_138615.2:c.1940A>G p.(Glu647Gly) | 1 | 0 | 3 | PM2, PP3 |
| *DJ1* | NM_007262.4:c.240G>T p.(Gln80His) | 1 | 0 | 3 | PM2 |
| *DJ1* | NM_007262.4:c.105dupT p.(Ala36Cysfs*12) | 1 | 3.98e-6 | 5 | PM3, PVS1, PM2, PP5 |
| *DJ1* | NM_007262.4:c.323G>C p.(Gly108Ala) | 1 | 0 | 4 | PM2, PM5, PP3 |
| *DJ1* | NM_007262.4:c.328A>G p.(Thr110Ala) | 1 | 9.90e-5 | 3 | PM2 |
| *DNAJC13* | NM_015268.4:c.5075T>C p.(Phe1692Ser) | 1 | 3.98e-6 | 3 | PM2, PP3, PP2 |
| *DNAJC13* | NM_015268.4:c.6457G>A p.(Ala2153Thr) | 1 | 8.97e-5 | 3 | PM2, PP2, BP4 |
| *DNAJC6* | NM_014787.3:c.595C>T p.(Arg199*) | 1 | 0 | 4 | PVS1, PM2 |
| *DNAJC6* | NM_001256864.1:c.2227+5A>C | 1 | 4.01e-6 | 3 | PM2 |
| *DNAJC6* | NM_001256864.1:c.1492T>A p.(Cys498Ser) | 1 | 1.31e-3 | 2 | BA1, BP6 |
| *EIF4G1* | NM_182917.4:c.1546C>T p.(Arg516Trp) | 1 | 2.83e-5 | 3 | PM2, PP2, BP4 |
| *FBXO7* | NM_012179.3: c.1220C>T p.(Pro407Leu) | 1 | 1.41e-5 | 3 | PM2 |
| *FBXO7* | NM_012179.3:c.1546G>C p.(Asp516His) | 1 | 1.60e-3 | 2 | BS1, BS2, BP6 |
| ***GBA1*** | **NM_001005741.2:c.1226A>G p.(Asn409Ser)** | **11** | **2.23e-3** | **5** | **PM1, PP2, PM2, PM5, PP3, PP5** |
| ***GBA1*** | **NM_000157.3 c.115+1G>A** | **1** | **7.09e-5** | **5** | **PS4, PVS1, PM2, PP5** |
| ***GBA1*** | **NM_000157.3:c.1342G>C p.(Asp448His)** | **5** | **1.28e-4** | **5** | **PM3, PS3, PM1, PP2, PM2, PM5, PP3, PP5** |
| ***GBA1*** | **NM_000157.3:c.370A>G p.(Met124Val)** | **1** | **7.07e-6** | **4** | **PM2, PP2** |
| *GBA1* | NM_000157.3:c.168C>T p.(Val56=) | 1 | 9.55e-5 | 2 | PM2, BP7, BP6 |
| ***GBA1*** | **NM_000157.3:c.413delC p.(Pro138Leufs*62)** | **1** | **0** | **4** | **PVS1, PM2, PP5** |
| ***GBA1*** | **NM_000157.3:c.1308dupT p.(Val437Cysfs*32)** | **1** | **0** | **4** | **PVS1, PM2** |
| ***GBA1*** | **NM_000157.4:c.850C>A p.(Pro284Thr)** | **1** | **3.19e-5** | **4** | **PM1, PP2, PM2, PP3, PP5** |
| ***GBA1*** | **NM_000157.4:c.754T>A (p.Phe252Ile)** | **1** | **4.24e-5** | **5** | **PM3, PS3, PM1, PP2, PM2, PP3, PP5** |
| ***GBA1*** | **NM_000157.4:c.1448T>C p.(Leu483Pro)** | **1** | **1.30e-3** | **5** | **PM3, PS3, PM1, PP2, PM2, PM5, PP3, PP5** |
| ***GBA1*** | **NM_000157.4:c.256C>T p.(Arg86*)** | **1** | **0** | **5** | **PS4, PVS1, PM2, PP5** |
| ***GBA1*** | **NM_000157.4:c.1093G>A p.(Glu365Lys)** | **1** | **1.07e-2** | **3** | **PP2, PP5** |
| ***GBA1*** | **NM_001005741.2:c.721G>A p.(Gly241Arg)** | **1** | **3.18e-5** | **5** | **PM3, PP1, PS3, PM1, PP2, PM2, PM5, PP3, PP5** |
| ***GBA1*** | **NM_000157.4:c.741delC p.(Trp248Glyfs*6)** | **1** | **0** | **4** | **PVS1, PM2** |
| ***GBA1*** | **NM_000157.4:c.604C>T p.(Arg202*)** | **1** | **8.81e-6** | **5** | **PM3, PVS1, PM2, PP5** |
| ***GBA1*** | **NM_001005741.2:c.882T>G p.(His294Gln)** | **1** | **2.19e-4** | **4** | **PS4, PM1, PP2, PM2, PP5** |
| *GRN* | NM_002087.4;c.1540G>A p.(Val514Met) | 1 | 4.60e-5 | 3 | PM2, BP4 |
| ***GRN*** | **NM_002087.2:c.813_816delCACT p.(Thr272Serfs*10)** | **1** | **0** | **5** | **PS4, PVS1, PM2, PP5** |
| *GRN* | NM_002087.2:c.1633C>T p.(Pro545Ser) | 1 | 0 | 3 | PM2, PP3 |
| *IMPDH2* | NM_000884.2:c.504G>C p.(Glu168Asp) | 1 | 7.95e-6 | 3 | PM2, PP2 |
| *KCNA1* | NM_000217.3:c.392G>A p.(Arg131Gln) | 1 | 0 | 3 | PM2, PP3 |
| *KCNMA1* | NM_001161352.1:c.3635C>T p.(Thr1212Ile) | 1 | 4.95e-5 | 3 | PM2, PP2 |
| *KCTD17* | NM_001282684.1:c.337G>A p.(Glu113Lys) | 1 | 1.60e-5 | 3 | PM2, PP3 |
| *KMT2B* | NM_014727.1:c.2218_2226delCCCCTGCAG p.(Pro740_Gln742del) | 1 | 0 | 3 | PM2, PM4 |
| *KMT2B* | NM_014727.3:c.2849G>A p.(Arg950Gln) | 1 | 4.02e-6 | 3 | PM2, BP4 |
| ***LRRK2*** | **NM_198578.3:c.4748T>G p.(Leu1583Arg)** | **1** | **0** | **4** | **PM2, PP3, PM6** |
| ***LRRK2*** | **NM_198578.3:c.4321C>T p.(Arg1441Cys)** | **1** | **1.19e-5** | **5** | **PS4, PP1, PS3, PM2, PM5, PP3, PP5** |
| ***LRRK2*** | **NM_198578.3:c.6055G>A p.(Gly2019Ser)** | **4** | **4.88e-4** | **5** | **PS4, PP3, PM2, PP5** |
| *LRRK2* | NM_198578.3:c.5800T>C p.(Ser1934Pro) | 1 | 0 | 3 | PM2, PP3 |
| *LRRK2* | NM_198578.3:c.7067C>T p.(Thr2356Ile) | 1 | 1.77e-4 | 3 | PM2, BP6 |
| ***LRRK2*** | **NM_198578.3:c.4322G>A p.(Arg1441His)** | **1** | **3.25e-4** | **4** | **PM2, PM5, PP5** |
| ***LRRK2*** | **NM_198578.4:c.5096A>G p.(Tyr1699Cys)** | **1** | **0** | **4** | **PM2, PP3, PP5, PP1** |
| ***LRRK2*** | **NM_198578.3:c.4321C>G p.(Arg1441Gly)** | **1** | **3.98e-6** | **5** | **PS4, PP1, PS3, PM2, PM5, PP3, PP5** |
| *LRRK2* | NM_198578.3:c.6010A>G p.(Thr2004Ala) | 1 | 7.96e-6 | 3 | PM2 |
| *LRRK2* | NM_198578.3:c.5606T>C p.(Met1869Thr) | 1 | 3.25e-4 | 3 | PM2 |
| ***LRRK2*** | **NM_198578.4: c.6059T>C p.(Ile2020Thr)** | **1** | **0** | **5** | **PS4, PP3, PM2, PP5** |
| *LRP10* | NM_014045.3:c.578A>G p.(Asn193Ser) | 1 | 3.18e-5 | 3 | PM2 |
| *LRP10* | NM_014045.3:c.1106C>T p.(Ser369Phe) | 1 | 3.59e-5 | 3 | PM2 |
| *MAPT* | NM_001377265.1:c.1342C>T p.(Arg448*) | 1 | 9.19e-5 | 3 | PM2, PVS1 |
| *NKX2-1* | NM_001079668.2:c.893A>T p.(Lys298Ile) | 1 | 0 | 3 | PM2, PP3 |
| *NPC1* | NM_000271.4:c.3689T>C p.(Leu1230Ser) | 1 | 2.39e-5 | 4 | PM2, PM1, PP2, PP3, PP5 |
| *NPC2* | NM_006432.5:c.58G>T p.(Glu20*) | 1 | 7.75e-5 | 5 | PM3, PVS1, PM2, PP5 |
| ***PRKN*** | **NM_004562.2: c.1296G>A p.(Met432Ile)** | **1** | **0** | **4** | **PM2, PP3, PM3** |
| ***PRKN*** | **NM_004562.2:c.823C>T p.(Arg275Trp)** | **4** | **1.97e-3** | **5** | **PM3, PP1, PS3, PM2, PP3, PP5** |
| *PRKN* | NM_004562.2:c.766C>T p.(Arg256Cys) | 1 | 4.28e-4 | 4 | PM2, PP3, PP5 |
| ***PRKN*** | **NM_004562.2:c.535_618del p.(Gly179_Ala206del)** | **1** | **0** | **4** | **PM2, PP5, PM3** |
| *PRKN* | NM_004562.2:c.1205G>A p.(Arg402His) | 1 | 7.16e-5 | 3 | PM2, PM5, PP3, PP5 |
| ***PRKN*** | **NM_004562.2:c.767G>A p.(Arg256His)** | **1** | **7.96e-6** | **4** | **PM3, PM2, PM5, PP3** |
| ***PRKN*** | **Exon 3 deletion** | **1** | **0** | **4** | **PM2, PM3** |
| *PRKN* | NM_004562.2:c.571C>T p.(Arg191Trp) | 1 | 5.66e-5 | 3 | PM2, PP3 |
| *PRKN* | NM_004562.2:c.1310C>T p.(Pro437Leu) | 1 | 1.57e-3 | 3 | BP6 |
| ***PRKN*** | **NM_004562.2:c.633A>T p.(Lys211Asn)** | **1** | **1.99e-5** | **4** | **PM3, PP1, PS3, PM2, PP3, PP5** |
| *PDE8B* | NM_003719.3:c.61G>C p.(Asp21His) | 1 | 0 | 3 | PM2, PP2 |
| *PINK1* | NM_032409.2:c.1366C>T p.(Gln456*) | 1 | 3.89e-5 | 5 | PM3, PVS1, PM2, PP5 |
| *PLA2G6* | NM_003560.2:c.391A>T p.(Ile131Phe) | 1 | 0 | 3 | PM2, PP2 |
| *PLA2G6* | NM_003560.2:c.1615G>A p.(Gly539Ser). | 1 | 7.04e-4 | 3 | PP2, BP6 |
| *PNKD* | NM_015488.4:c.585delG p.(Ser196Alafs*77) | 1 | 3.70e-5 | 3 | PM2 |
| *POLG* | NM_002693.3:c.1760C>T p.(Pro587Leu) cis | 1 | 1.54e-3 | 4 | PS4, PM1, PP2, PM2, PP3, PP5 |
| *POLG* | NM_002693.3:c.752C>T p.(Thr251Ile) cis | 1 | 1.54e-3 | 4 | PS4, PM2, PP2, PP5 |
| ***POLG*** | **NM_002693.2:c.2864A>G p.(Tyr955Cys)** | **1** | **0** | **5** | **PM3, PP1, PS3, PM1, PP2, PM2, PP3, PP5** |
| *PRRT2* | NM_001256442.1:c.649dupC p.(Arg217Profs*8) | 1 | 0 | 5 | PP1, PS3, PVS1, PS2, PP5 |
| *SCN2A* | NM_001040142.1:c.2119A>T p.(Ile707Leu) | 1 | 1.19e-5 | 3 | PM2, PP2, BP6 |
| *SLC2A1* | NM_006516.4:c.19-5C>T | 1 | 7.89e-5 | 2 | PM2, BP4 |
| *SNCA* | NM_007308.2:c.50C>A p.(Ala17Asp) | 1 | 0 | 3 | PM2, PP3 |
| *SYNE1* | NM_182961.3:c.650T>C p.(Ile217Thr) | 1 | 3.98e-6 | 3 | PP3, PM2 |
| *SYNE1* | NM_182961.3:c.17683G>A p.(Asp5895Asn) | 1 | 3.99e-6 | 3 | PM2, PP2, BP4 |
| *TWNK* | NM_001199135.3:c.1159G>A p.(Asp387Asn) | 1 | 0 | 3 | PM2, BP4 |
| *UCHL1* | NM_004181.5:c.592G>A p.(Ala198Thr) | 1 | 1.07e-5 | 3 | PM2 |
| *VPS13C* | NM_020821.2:c.8996T>C p.(Met2999Thr) | 1 | 7.97e-5 | 3 | PM2, BP4 |
| *VPS13C* | NM_020821.2:c.9909dupT p.(Ile3304Tyrfs*2) | 1 | 3.19e-5 | 4 | PVS1, PM2 |
| *VPS13C* | NM_020821.2:c.2797A>G p.(Thr933Ala) | 1 | 3.58e-3 | 2 | BA1, BS2, BP4, BP6 |
| *VPS13C* | NM_020821.2:c.9535A>G p.(Ile3179Val) | 1 | 0 | 3 | PM2 |
| *VPS13C* | NM_020821.2:c.1748A>G p.(Tyr583Cys) | 1 | 4.39e-5 | 3 | PM2 |
| *VPS13C* | NM_020821.2:c.3515C>T p.(Thr1172Ile) | 1 | 3.59e-5 | 3 | PM2, BP4 |
| *VPS13C* | NM_020821.2:c.9526C>G p.(Arg3176Gly) | 1 | 1.78e-3 | 2 | BA1, BS2, BP4, BP6 |
| *VPS13C* | NM_020821.2:c.395C>G p.(Ser132*) | 1 | 3.98e-6 | 4 | PVS1, PM2 |
| *VPS13C* | NM_020821.2:c.7063-4A>G | 1 | 0 | 3 | PM2 |
| ACMG is reported as calculated by FranklinGenoox and Varsome; MAF: maximum allele frequency in GnomAD 2.1; N: number of cases in the cohort | | | | | |

**Supplementary Table III:** List of patients with a definite genetic diagnosis and their gene variants

| ***Patient’s code number*** | ***Gene*** | ***Variant*** | ***Zigosity**** |
| --- | --- | --- | --- |
| 6 | *GBA1* | NM_001005741.2:c.721G>A p.(Gly241Arg) | E |
| 19 | *GBA1* | NM_000157.4:c.1342G>C p.(Asp448His) | E |
| 30 | *PRKN* | Exon 3 deletion | E |
|  | *PRKN* | NM_004562.2:c.767G>A p.(Arg256His) | E |
| 34 | *GBA1* | NM_000157.3:c.1308dupT p.(Val437Cysfs*32) | E |
| 36 | *GBA1* | NM_001005741.2:c.1226A>G p.(Asn409Ser) | E |
| 37 | *GBA1* | NM_001005741.2:c.1226A>G p.(Asn409Ser) | E |
| 38 | *GBA1* | NM_001005741.2:c.1226A>G p.(Asn409Ser) | E |
| 39 | *GBA1* | NM_000157.3 c.115+1G>A | E |
| 40 | *GBA1* | NM_001005741.2:c.1226A>G p.(Asn409Ser) | E |
| 41 | *GBA1* | NM_000157.3:c.1342G>C p.(Asp448His) | E |
| 42 | *GBA1* | NM_000157.3:c.370A>G p.(Met124Val) | E |
|  | *GBA1* | NM_000157.3:c.168C>T p.(Val56=) | E |
| 43 | *GBA1* | NM_001005741.2:c.1226A>G p.(Asn409Ser) | E |
| 44 | *GBA1* | NM_001005741.2:c.1226A>G p.(Asn409Ser) | E |
| 45 | *GBA1* | NM_000157.3: c.1342G>C p.(Asp448His) | E |
| 46 | *GBA1* | NM_000157.3:c.413delC p.(Pro138Leufs*62) | E |
| 47 | *GBA1* | NM_001005741.2:c.1226A>G p.(Asn409Ser) | E |
| 48 | *GBA1* | NM_000157.4:c.1342G>C p.(Asp448His) | E |
| 49 | *GBA1* | NM_000157.4:c.1342G>C p.(Asp448His) | E |
| 50 | *GBA1* | NM_001005741.2:c.1226A>G p.(Asn409Ser) | E |
| 51 | *GBA1* | NM_000157.4:c.850C>A p.(Pro284Thr) | E |
| 52 | *GBA1* | NM_000157.4:c.754T>A (p.Phe252Ile) | E |
| 53 | *GBA1* | NM_001005741.2:c.1226A>G p.(Asn409Ser) | E |
|  | *GBA1* | NM_000157.4:c.1448T>C p.(Leu483Pro) | E |
| 54 | *GBA1* | NM_000157.4:c.256C>T p.(Arg86*) | E |
| 55 | *GBA1* | NM_000157.4:c.1093G>A p.(Glu365Lys) | E |
| 56 | *GBA1* | NM_001005741.2:c.1226A>G p.(Asn409Ser) | E |
| 57 | *GBA1* | NM_001005741.2:c.1226A>G p.(Asn409Ser) | E |
| 58 | *GBA1* | NM_000157.4:c.741delC p.(Trp248Glyfs*6) | E |
| 59 | *GBA1* | NM_000157.4:c.604C>T p.(Arg202*) | E |
| 60 | *GBA1* | NM_001005741.2:c.882T>G p.(His294Gln) | E |
| 62 | *GRN* | NM_002087.2:c.813_816delCACT p.(Thr272Serfs*10) | E |
| 65 | *LRRK2* | NM_198578.3:c.6055G>A p.(Gly2019Ser) | E |
| 68 | *LRRK2* | NM_198578.3:c.4748T>G p.(Leu1583Arg) | E |
| 69 | *LRRK2* | NM_198578.3:c.4321C>T p.(Arg1441Cys) | E |
| 70 | *LRRK2* | NM_198578.3:c.6055G>A p.(Gly2019Ser) | E |
| 73 | *LRRK2* | NM_198578.3:c.4322G>A p.(Arg1441His) | E |
| 74 | *LRRK2* | NM_198578.3:c.6055G>A p.(Gly2019Ser) | E |
| 75 | *LRRK2* | NM_198578.3:c.6055G>A p.(Gly2019Ser) | E |
| 76 | *LRRK2* | NM_198578.4:c.5096A>G p.(Tyr1699Cys) | E |
| 77 | *LRRK2* | NM_198578.3:c.4321C>G p.(Arg1441Gly) | E |
| 78 | *LRRK2* | NM_198578.4: c.6059T>C p.(Ile2020Thr) | E |
| 83 | *PRKN* | NM_004562.2: c.1296G>A p.(Met432Ile) | O |
| 87 | *PRKN* | NM_004562.2:c.823C>T p.(Arg275Trp) | O |
| 88 | *PRKN* | NM_004562.2:c.535_618del p.(Gly179_Ala206del) Exon 5 deletion | O |
| 90 | *PRKN* | NM_004562.2:c.633A>T p.(Lys211Asn) | E |
|  | *PRKN* | NM_004562.2:c.823C>T p.(Arg275Trp) | E |
| 96 | *POLG* | NM_002693.2:c.2864A>G p.(Tyr955Cys) | O |

**Supplementary Table III:** List of GBA variants found and their relative frequency in our study.
